# Synthesis of Hierarchical Zeolites with Morphology Control: Plain and Hollow Spherical Beads of Silicalite-1 Nanosheets

**DOI:** 10.3390/molecules25112563

**Published:** 2020-05-31

**Authors:** Kassem Moukahhal, Bénédicte Lebeau, Ludovic Josien, Anne Galarneau, Joumana Toufaily, Tayssir Hamieh, T. Jean Daou

**Affiliations:** 1 University of Haute Alsace (UHA), CNRS, Axe Matériaux à Porosité Contrôlée (MPC), Institut de Science des Matériaux de Mulhouse (IS2M), UMR 7361, F-68093 Mulhouse, France; kassem.moukahhal@uha.fr (K.M.); benedicte.lebeau@uha.fr (B.L.); ludovic.josien@uha.fr (L.L.); 2 University of Strasbourg (UniStra), F-67000 Strasbourg, France; 3 Laboratory of Materials, Catalysis, Environment and Analytical Methods Faculty of Sciences, Section I, Lebanese University Campus Rafic Hariri, Hadath, Lebanon; joumana.toufaily@ul.edu.lb (J.T.); tayssir.hamieh@ul.edu.lb (T.H.); 4 ICGM, University of Montpellier, CNRS, ENSCM, 34296 Montpellier, France; anne.galarneau@enscm.fr

**Keywords:** zeolites, zeosils, nanosheets, hierarchical zeolite, binderless zeolite, MFI, pollutant decontamination, volatile organic compounds, air purification

## Abstract

Binderless pure silica zeolites (zeosils) spheres and hollow spheres with a diameter of 20 µm composed of silicalite-1 nanosheets particles were prepared by pseudomorphic transformation of spherical silica beads using different temperatures (110, 130, and 150 °C) and treatment times (1–5 days) in order to adapt the local dissolution rate of silica to the crystallization rate of silicalite-1 nanosheets allowing to preserve the initial morphology of the silica beads. Fully crystalline beads of 20 µm were obtained at 110 °C for 5 days, whereas hollow spheres similar in size were synthesized at higher temperatures. The crystallization process seems to begin at the outer surface of the amorphous silica beads and spreads with the time in the interior of the beads leading to a dissolution of the inner amorphous part of the beads to create zeosil hollow spheres for the highest treatment temperatures (130 and 150 °C). The dissolution rate of the inner amorphous part of the beads increases by increasing the hydrothermal treatment temperature from 130 to 150 °C. The silicalite-1 beads synthesized at 110 °C for 5 days showed to be promising for rapid molecular decontamination by adsorbing n-hexane in larger amount than the silicalite-1 conventional big crystals in powder forms.

## 1. Introduction

Pure silica zeolites known under the name of zeosils are microporous materials with enhanced mechanical and thermal properties compared to aluminosilicate zeolites [1,2,3,4,5]. Due to their hydrophobic character, zeosils are excellent candidates for mechanical energy storage [3,4,5] and molecular decontamination [1,2,6,7,8]. In the case of molecular decontamination, these materials have the supplementary advantage on aluminosilicate zeolites that their volatile organic compounds (VOCs) sorption properties are not hindered by the significant quantity of water present in the air [1,6].

However, conventional syntheses of zeolites give often rise to powders composed of micron-size crystals. Unfortunately, those powders cannot be used directly for industrial applications [9], especially for molecular decontamination because secondary dust contamination due to zeolite particles spreading could occurs [8,10,11,12,13,14,15]. The conventional micron-size crystals have also the disadvantages to induce some diffusional limitations. In order to overcome these constraints, shaping of hierarchical zeolite materials (beads, monoliths, etc.) composed of nanocrystals is needed. This synthesis strategy has the advantage to give a usable product for molecular decontamination, which avoids diffusional problems and possesses higher porous volume (due to the introduction of additional porosity) and therefore adsorption properties than conventional zeolites.

Zeolite microspheres with hierarchical porosities are emerging as attractive materials for applications in adsorption and catalysis [11,12,16,17,18]. Hollow zeolite spheres have been fabricated by assembly of nano-zeolites into macroscopic structures and removal of templates [19,20,21,22].

Zeolite microspheres composed of small crystals were prepared by using templates, which, upon removal, determine the pore structure of the products. Wang et al. [23] developed a new method for the preparation of silicalite-1 microspheres using impregnated monodispersed micron-sized poly-styrene-*co*-divinylbenzene porous particles as template. Yin et al. [24] used dimethyldiallyl ammonium chloride acrylamide copolymer as a template for fast and one-step formation of nano-zeolite beta microspheres. Tao et al. [25] reported space-confined synthesis route of hierarchical MFI zeolite microspheres respectively with nanorod oriented-assembled structures of a carbon–silica composite monolith via hydrothermal treatment. Wang et al. [26] hydrothermally synthesized hierarchical ZSM-5 zeolite microspheres by using organo-functionalized silanized mesoporous silica as silica source. The preparation of spherical macrostructures employing anion exchange resin beads as templates was also reported by Tosheva et al. [27,28], Yin et al. [29], and our team [16] for MFI, *BEA and LTA-type zeolites. Zeolite beads with tuned size were also elaborated in two steps thanks to a shearer/mixer in the presence of carboxymethylcellulose, sodium metasilicate, or clays as binders [11,12].

The above reported methods have the disadvantage of using a shape directing templates or binders. Therefore, simple and binderless methods to synthesize hierarchical shaped zeolite are required such as pseudomorphic transformation [3,4,5,6,7,8,9,10,11,12,13,14,15,16,17,18,19,20,21,22,23,24,25,26,27,28,29,30].

By exploiting the concept of pseudomorphic transformation, i.e., using silica amorphous beads or monoliths featuring mesopores as silica source, the group of A. Galarneau was able to shape SOD, LTA, FAU (13X) zeolites, featuring an Si/Al ratio close to 1, as beads of 10 µm, 60 µm, and 1 mm [18] and as monoliths of 0.6 cm diameter and 3 cm length with hierarchical porosity (micro-/meso-/macroporosity) [31,32] without binders. The zeolite beads have the same size and shape as the initial amorphous silica beads used in the pseudomorphic synthesis. Recently, we succeeded to obtain hierarchical MFI zeolite beads as ZSM-5 with Si/Al ratio around 40 by the pseudomorphic synthesis technology [33]. Different diameters of beads were obtained (20, 50, and 75 µm) and each bead was built by the assembly of ZSM-5 zeolite nanosheets of 2 nm thickness. To our knowledge, no studies involving pseudomorphic transformation, has been reported for the direct synthesis of pure silica MFI zeolite beads composed of small silicalite-1 nanocrystals with hierarchical porosity involving inter-crystalline mesopores.

Silicalite-1 zeosil belong to MFI-structure type, which is characterized by a porous network formed by the interconnection of straight circular channels (5.4 Å × 5.6 Å) with sinusoidal and elliptical channels (5.1 Å × 5.4 Å) [1,2,3,4,5,34]. The latter structure is of particular interest for several environmental applications such as energy storage [3,4,5] and air purification as VOCs adsorption [1,2,6].

This work will therefore address the synthesis of hierarchical silicalite-1 zeolite beads and hollow spheres composed of zeosil nanosheets using amorphous silica beads of 20 µm in size as starting material for the pseudomorphic transformation. The effect of hydrothermal treatment time and temperature on the pseudomorphic synthesis of pure and well-crystallized silicalite-1 beads and hollow spheres and their ability to adsorb n-hexane, a well-known VOC, will be discussed.

## 2. Results and Discussion

**Figure 1:**
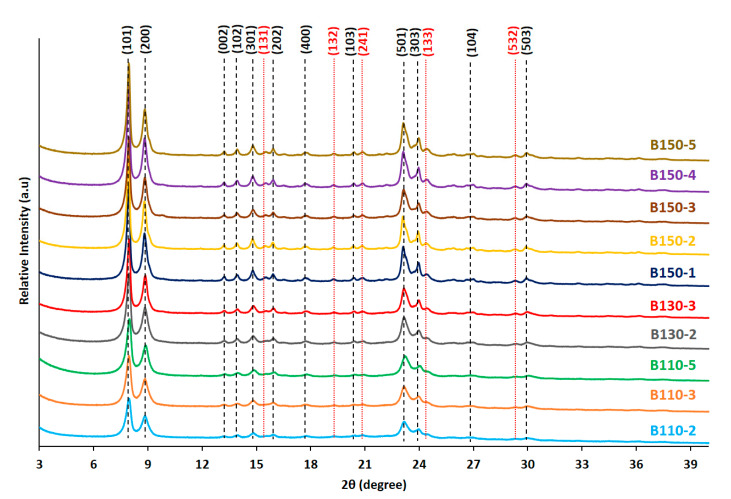
X-ray diffraction (XRD) patterns of calcined silicalite-1 beads (B-T(°C)-t(days)) obtained at different temperatures and treatment times.

The crystallinity and purity of the synthesized silicalite-1 beads obtained by pseudomorphic transformation of silica beads using typical diquaternary ammonium surfactant C22-6-6 to produce MFI nanosheets were first checked by X-ray diffraction (XRD). According to the XRD patterns reported in Figure 1, the materials obtained after 2 or 3 days of synthesis at 110 °C (B110-2 and B110-3) contains MFI-type zeolite (silicalite-1) with some amorphous materials. In order to avoid the presence of amorphous phase, the syntheses were then conducted at higher crystallization time (5 days) or higher temperatures, 130 and 150 °C. When the synthesis time at 110 °C was increased to 5 days or the synthesis temperature was increased to 130 and 150 °C, pure silicalite-1 zeolite samples were obtained. For all these samples, the XRD diffraction peaks are less intense than those of the conventional nanosheet silicalite-1 featuring crystal size of several micron [2,3]. Using amorphous silica beads as silica source for the synthesis of silicalite-1 zeolite beads composed of nanosheets seems to accelerate the kinetic of crystallization compared to the conventional synthesis of silicalite-1 nanosheets powder using tetraethylorthosilicate (TEOS) as silica source (as mentioned in our previous paper) [2]. With TEOS 10 days of crystallization at 110 °C were needed to obtain fully crystallized MFI-type nanosheets exempt of amorphous phase, whereas only 5 days were enough with silica beads for the same synthesis temperature. Moreover, all the diffraction peaks observed on the XRD patterns of the samples synthesized at 110 and 130 °C belong to the crystallographic plane (h0l), providing the sign that a one-dimension growth-inhibition is undertaken along the *b*-axis (2 nm of thickness), thus leading to the formation of nanosheets [2,3,35,36]. When the synthesis temperature was increased up to 150 °C additional (hkl) (with k ≠ 0) peaks with low intensities corresponding also to MFI-type zeolite structure are detected indicating the presence of bigger crystallites or higher thickness of the sheets.

**Figure 2:**
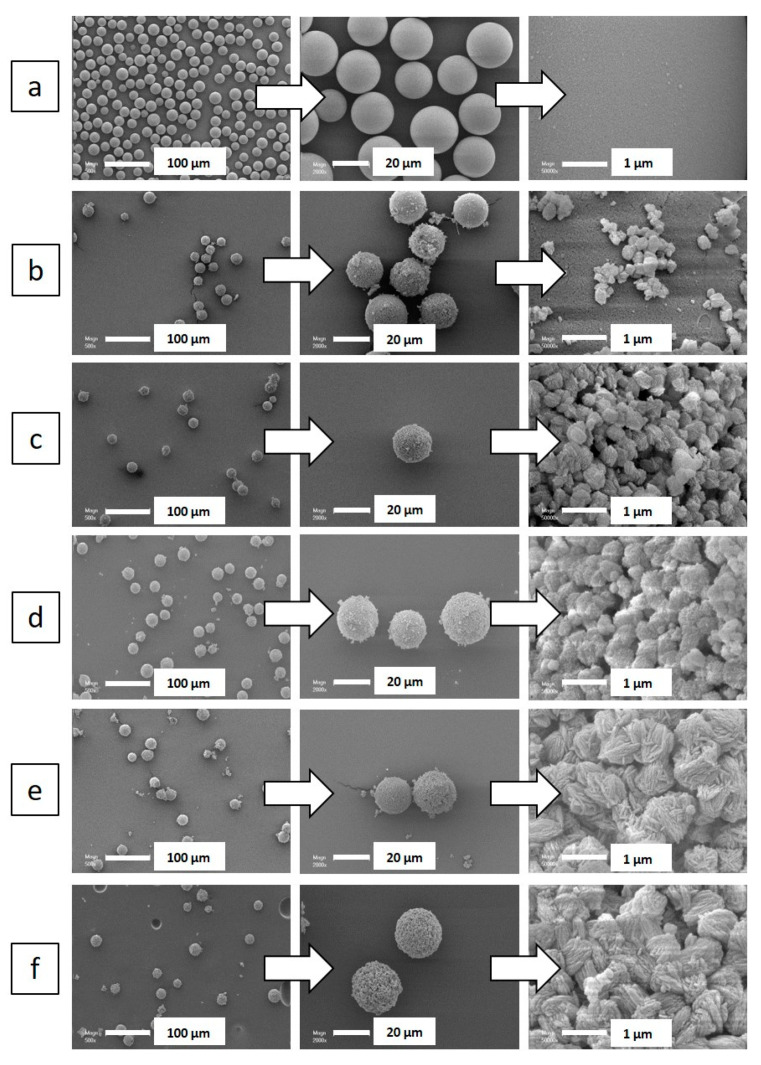

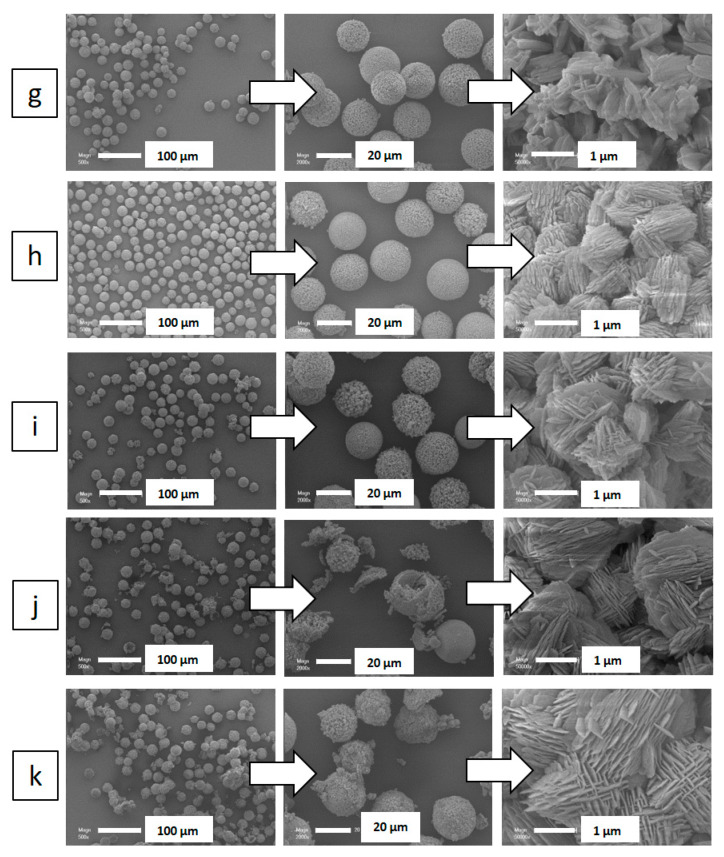
Scanning electron microscopy (SEM) images of: (**a**) amorphous 20 µm silica spheres, and silicalite-1 beads as a function of their temperature and duration synthesis (B-T(°C)-t(days)): (**b**) B110-2, (**c**) B110-3, (**d**) B110-5, (**e**) B130-2, (**f**) B130-3, (**g**) B150-1, (**h**) B150-2, (**i**) B150-3, (**j**) B150-4, and (**k**) B150-5.

Scanning electron microscopy (SEM) images displayed in Figure 2 show the conservation of the initial spherical morphology and size (20 µm) of the amorphous silica beads. The silicalite-1 beads are composed of an agglomeration of zeolite particles of 0.2–2.8 μm diameter (Table 1), each composed of silicalite-1 nanosheets. The size of the silicalite-1 particles (resulting from nanosheets agglomeration) seems to increase by increasing the hydrothermal treatment time and temperature (Table 1).

**Figure 3:**
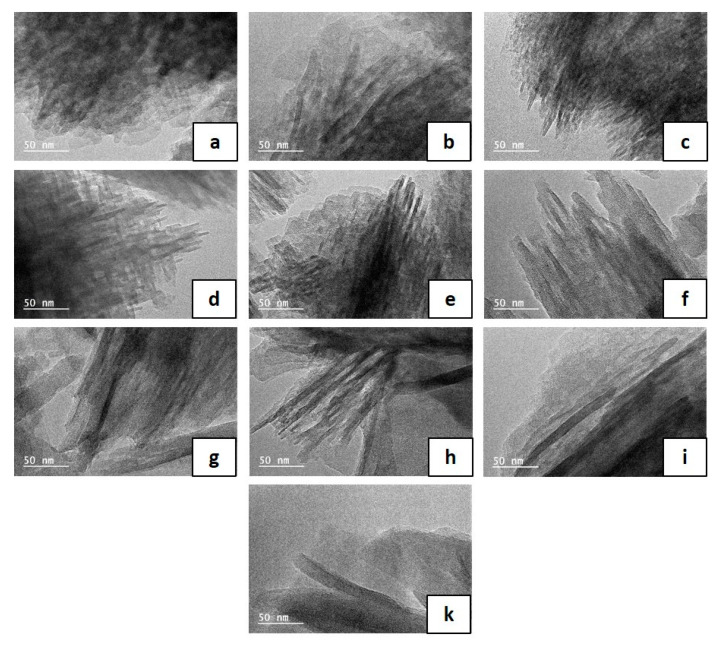
Transmission electron microscopy (TEM) images of silicalite-1 beads as a function of their temperature and duration synthesis (B-T(°C)-t(days)): (**a**) B110-2, (**b**) B110-3, (**c**) B110-5, (**d**) B130-2, (**e**) B130-3, (**f**) B150-1, (**g**) B150-2, (**h**) B150-3, (**i**) B150-4, and (**j**) B150-5.

**Figure 4:**
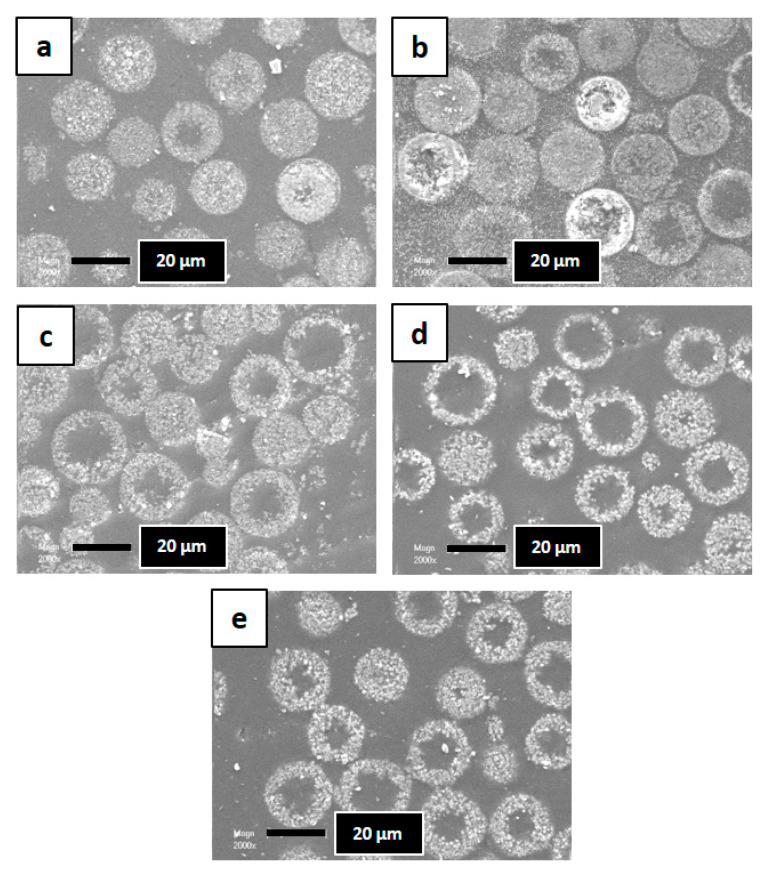
SEM images of the synthesized silicalite-1 beads embedded in polymer and grinded. Silicalite-1 beads are presented as a function of their temperature and duration synthesis (B-T(°C)-t(days)): (**a**) B110-3, (**b**) B110-5, (**c**) B130-2, (**d**) B150-1, and (**e**) B150-5.

Transmission electron microscopy (TEM) images given in Figure 3 show that the silicalite-1 nanosheets composing the beads obtained after a hydrothermal treatment temperature of 110 and 130 °C have a thickness of 2 nm. An increase of the nanosheet size (4–8 nm) composing the beads is observed for hydrothermal treatment at 150 °C (Table 1). Increasing the duration of the hydrothermal treatment at 150 °C seems also to increase also the nanosheet thickness from 4 to 8 nm (Table 1). This is consistent with XRD data, where additional (hkl) (with k ≠ 0) peaks with low intensities are observed for these samples. This phenomenon could be explained by “Ostwald ripening” which is usually observed in inorganic synthesis where small crystals dissolve to generate the growth of big crystals and/or the dissolution of inner amorphous silica of the beads because pseudomorphic transformation is known to begin at the outer surface of the samples and diffuse with time to the inner part of the samples [33]. In order to see the evolution of the interior part of the beads as function of the treatment temperature and time, SEM images (Figure 4) of the synthesized samples embedded in polymer and then grinded were realized. The crystallization seems to begin at the outer surface of the beads. While further increasing the hydrothermal temperature (130 and 150 °C), the dissolution kinetic of the inner amorphous part of the beads seems to increase allowing the formation of a majority of hollow spheres instead of a majority of fully crystallized beads obtained at 110 °C for 5 days of treatment (sample B110-5).

**Figure 5:**
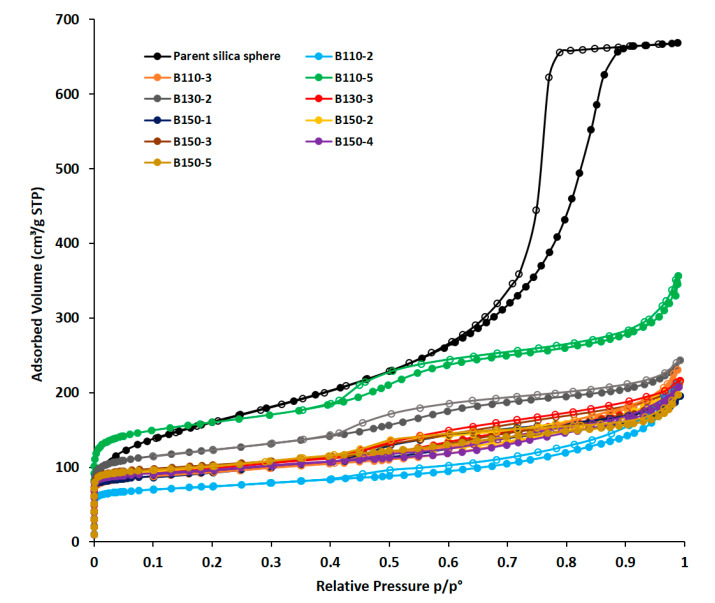
N_2_ adsorption–desorption isotherms at −196 °C of the calcined silicalite-1 beads obtained by pseudomorphic transformation at different temperatures (T) and durations (time) (B-T(°C)-time (days)) compared to the parent silica bead.

The textural properties of the synthetized silicalite-1 beads were studied by N_2_ sorption isotherm at −196 °C. Figure 5 shows the isotherms of the different calcined silicalite-1 beads obtained by pseudomorphic transformation in comparison to the parent amorphous silica beads. T-plot analysis [37] have been performed (Figure S1) and their textural properties are summarized in Table 2. The underestimation of microporous volumes and the overestimation of surface areas determined from the classical t-plot analysis have been corrected by using the abacus proposed by Galarneau et al. [37] for hierarchical zeolites featuring microporous volume to the total pore volume ratio (V_micro_ *_(t-plot)_*/V_tot *(t-plot)*_ %) higher than 20%.

**Figure 6:**
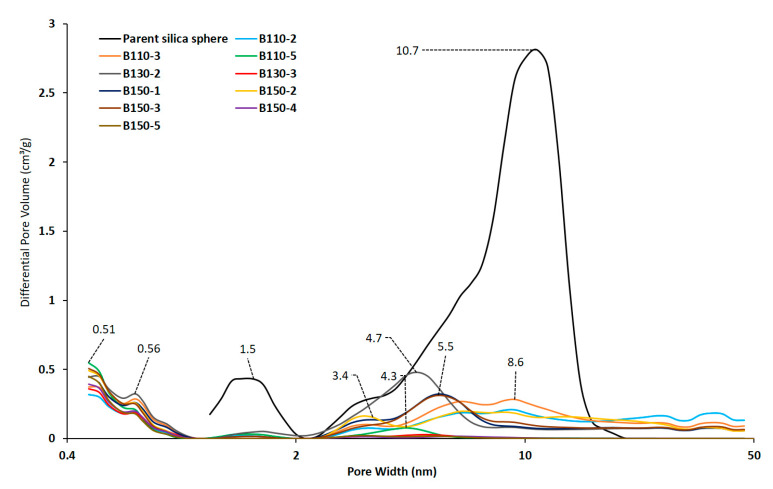
DFT (Density Functional Theory) pore size distributions determined from the adsorption branch of the N_2_ isotherms of calcined silicalite-1 beads obtained by pseudomorphic transformation at different temperatures (T) and durations (time in days) (B-T(°C)-t(days)) compared to the parent silica bead.

All of the isotherms are type I according to IUPAC [38] at low relative pressures and type II-b with hysteresis at high relative pressures. The presence of a hysteresis in the relative pressure range 0.4 < p/p° < 1 is typical of lamellar materials, due to the stacking of the nanosheets. The comparison with the isotherm of the parent 20 µm amorphous silica bead shows that the capillary condensation steps occur at a different relative pressure indicating that these small mesopores are formed during the crystallization of silicalite-1 nanosheets. However, since the distribution of mesopore size for parent 20 µm beads is wide and overlapping those of transformed beads it is not excluded that residual mesoporosity from parent beads remains as observed from the mesopores diameters determined from DFT (Density Functional Theory) for samples B110-2 and B110-3. From DFT pore size distributions of transformed beads and hollow spheres shown in Figure 6, micropores characteristics of MFI zeolite with sizes of 0.51 and 0.56 nm are observed. The crystallization degrees of silicalite-1 nanosheets beads and hollow spheres obtained by pseudomorphic transformation were thus estimated by comparing the microporous volumes corresponding to zeolitic micropores with the one of well crystallized MFI-type zeolite (0.18 mL/g). For the silicalite-1 beads synthesized at 110 °C, an increase in the zeolitic microporous volume and the degree of crystallization is observed while increasing the hydrothermal treatment time from 2 to 5 days. The low crystallization degree of samples B110-2 and B110-3 is in agreement with the presence of an amorphous phase observed by XRD. When comparing the samples obtained after 2 days of hydrothermal treatment but at different temperatures, the crystallization degree seems to increase by increasing the hydrothermal treatment temperature from 110 to 150 °C: 58% for sample B110-2, 86% for sample B130-2, and 90% for sample B150-2. The same trend is observed for the samples synthesized after 3 days of hydrothermal treatment but the increase of the crystallization degree imposed by the increase of the treatment temperature is less significant at 3 days compared to 2 days. When the hydrothermal time was increased to 5 days, the trend is inversed and the best crystallization degree (100%) is found for sample B110-5 obtained after 5 days at 110 °C. This result could be explained by the fact that at higher treatment temperature and longer treatment time the dissolution of amorphous silica and “Ostwald ripening” are higher. The sample with the highest micropore volume, the highest mesopore volume and the highest surface areas (micro, meso-, and external) is the silicalite-1 beads obtained at 110 °C for 5 days (B110-5) (Table 2). However, the obtained hollow spheres (samples obtained at 130 and 150 °C) are also promising materials for rapid processes under high flow rate and the sample obtained at 130 °C for 2 days (B130-2) appears to have the highest mesopore volume for similar micropore volumes and the highest micropore surface area and mesoporous and external surface area. These two silicalite-1 samples are good candidates to be tested in adsorption flow processes of air pollutants.

**Figure 7:**
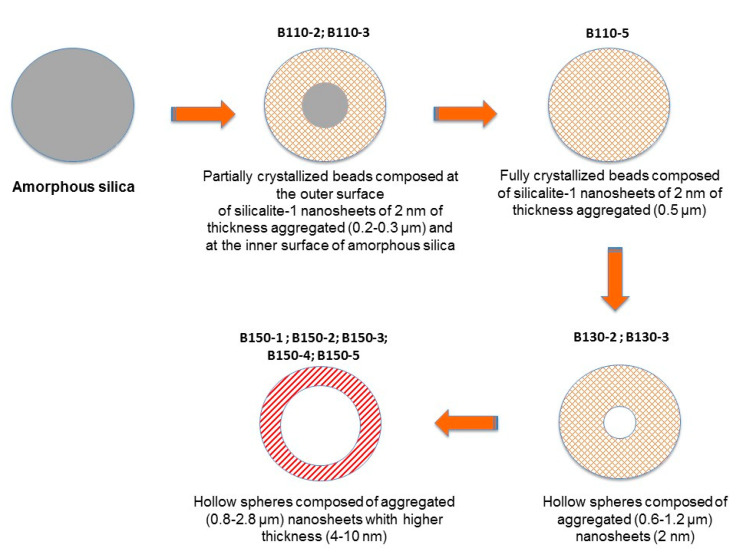
Schematic representation of the pseudomorphic transformation of silica amorphous beads into silicalite-1 beads or hollow spheres composed of silicalite-1 nanosheets samples (B-T(°C)-t(days)) as a function of the hydrothermal temperature (T in °C) and duration (t in days).

According to the results of textural and structural characterization shown above, a mechanism is proposed in Figure 7. Independently from the temperature and the treatment time, the crystallization seems to begin at the outer surface of the beads. While further increasing the hydrothermal temperature, the dissolution kinetic of the inner amorphous part of the beads seems to increase allowing the formation of a majority of hollow spheres instead of a majority of fully crystallized beads obtained at 110 °C for 5 days of treatment. Taking into account this information, and knowing from the TEM images that the length of silicalite-1 nanosheets is about 2 nm for the sample synthesized at 110 and 130 °C, and that this thickness seems to increase at 150 °C up to 8 nm a mechanism is proposed in Figure 7.

**Figure 8:**
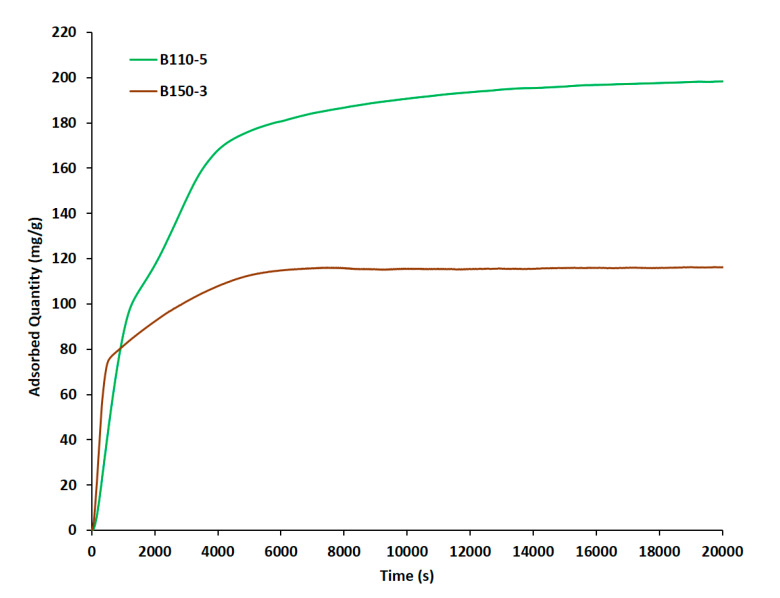
Adsorption kinetic of n-hexane at 25 °C and p/p° = 1 with silicalite-1 beads synthesized at 110 °C for 5 days (B110-5) and hollow spheres synthesized at 150 °C for 3 days (B150-3).

The *n-*hexane sorption capacity of the calcined conventional silicalite-1 zeosil at 25 °C is expected to be around 111–120 mg/g which corresponds to 7.4–8 molecules of n-hexane per MFI unit cell [1,2,6,39]. B110-5 and B150-3 showed a sorption capacity around 197 and 115 mg/g, respectively (which corresponds to 13.4 and 7.7 molecules of n-hexane per MFI unit cell). B110-5 sample is a highly promising material because it possesses higher adsorption capacities compared to conventional silicalite-1 microcrystals. The enhanced uptake rate of the n-hexane in B110-5 sample must be attributed to an improved accessibility, to a shorter diffusion path length in the micropores and to the presence of additional mesoporous volume. No loss of n-hexane adsorption capacity is observed for B110-5 compared to what is usually obtained for silicalite-1 nanosheets in powder form [2]. The adsorption curves in Figure 8 were reproduced three times over each sample. Hollow spheres as (B150-3) show highest initial rate of adsorption and might be promising for processes with higher flow rate. All hollow spheres should be tested.

## 3. Materials and Methods

### 3.1. Preparation of Silicalite-1 Beads

#### 3.1.1. Structure Directing Agent

The di-quaternary ammonium-type surfactant used for the transformation of amorphous silica spheres into MFI-type nanosheets spheres, ([C_22_H_45_–N^+^(CH_3_)_2_–C_6_H_12_–N^+^(CH_3_)_2_–C_6_H_13_]Br_2_) named (C_22-6-6_) was obtained in two steps following the procedure reported by Choi et al. [40] The surfactant was composed of a long-chain alkyl group (C_22_) and two quaternary ammonium groups spaced by a C_6_ alkyl linkage.

#### 3.1.2. Pseudomorphic Synthesis of Silicalite-1 Nanosheet Spheres

Pseudomorphic transformation were performed using as silica source, amorphous porous silica spheres Silica*Sphere*^TM^ delivered by *Silicycle^®^*. These mesoporous silica beads have sizes of 20 µm and mean pore diameters of 6 or 8 nm (data given by the supplier). 0.4 g of Sodium hydroxide (Carlo Erba, Val de Reuil, France, 99%) is dissolved in 12 mL deionized water in a beaker. Then 1.35 g of C_22-6-6_ and 0.31 g of sulfuric acid (Sigma-Aldrich, Saint Louis, MO, USA, 96%) were then added under stirring. After homogenization, the solution is transferred in a 45 mL Teflon*^®^*-lined stainless steel autoclave containing 1 g of amorphous silica spheres with 20 µm size to set the molar composition of the gel to: 1 SiO_2_: 0.3 Na_2_O: 0.18 H_2_SO_4_: 0.1 C_22-6-6_: 40 H_2_O. The autoclave was placed in an oven for various temperature and heating time in static mode.

After synthesis the product was recovered by filtration, washed with water and dried overnight at 80 °C. The C_22-6-6_ surfactant was removed by calcination in a muffle furnace at 550 °C at a rate of 1 °C/min for 8 h. The resulting zeolite beads will be named (B-T(°C)-t(days)), where “T” stands for the hydrothermal treatment temperature and “t” stands for the duration of the hydrothermal treatment in days.

### 3.2. Characterization of Zeosils

The purity and the crystallinity of the calcined silicalite-1 microspheres were checked by XRD analysis. X-ray diffraction patterns of the different materials introduced in a glass capillaries were recorded using a STOE STADI-P diffractometer (STOE δ Cie GmbH, Darmstadt, Germany) operating with Cu Kα_1_ radiation (λ = 0.15406 nm) in the range 3 < 2θ < 40 °.

The size and the morphology of the calcined zeosil spheres were determined by scanning electron microscopy (SEM) (an average was obtained by measuring 100 particles for each sample) using a Philips XL 30 FEG (Field Emission Gun) microscope (Verdun, France). The size of the silicalite-1 nanosheet crystals composing the spheres (an average was obtained by measuring the thickness of 100 nanosheets for each sample) was determined using a transmission electron microscopy (TEM) JEOL (Val de Reuil, France) model ARM-200F, under an acceleration voltage of 200 kV, with a point-to-point resolution of 80 pm. In order to detect the hollow presence or not in the inner part and the homogeneity of the beads with SEM, samples were embedded in cold mounting epoxy type resin (Struers, Epofix, Champigny sur Marne, France). Embedded beads were then grinded with various SiC grinding papers and finally polished with diamond polishing suspension (particle size 1 µm) until obtaining a soft surface.

Nitrogen adsorption/desorption isotherms at −196 °C were measured using a Micromeritics ASAP 2420 apparatus (Micromeritics, Merignac, France). Prior to the adsorption measurements, the calcined samples were outgassed for 1 h at 90 °C and then at 300 °C overnight under vacuum. The microporous volumes were determined from t-plot method by applying when needed a correction according to Galarneau et al. [37]. Mesoporous volume was found by subtracting the microporous volume from the total porous volume. The pore diameter distributions were obtained from the adsorption branch by applying DFT method that is reliable over the complete range of micro- and mesopores [38,41].

### 3.3. Dynamic n-Hexane Adsorption Measurements

Dynamic adsorption measurements were performed under VOC atmosphere (n-hexane) at 25 °C and controlled value of relative pressure p/p° = 1 (p is the vapor pressure and p_0_ is the saturation vapor pressure of n-hexane at 25 °C (p° = 202 hPa) using a thermogravimetric TG92 instrument (Setaram, Caluire et Cuire, France) [1,2,36]. The experiments were done under N_2_ flow. The gas flow rate was stable (114 mL/min). The experiment begun with an activation phase: the zeolites were heated up to 350 °C under dried nitrogen for 2 h, at atmospheric pressure to remove all adsorbate traces. Then, the sample was cooled to 25 °C, and the organic compound was introduced to the system. The adsorbed amount was then measured every 20 s.

## 4. Conclusions

This work highlights the pseudomorphic transformation of silica amorphous beads (20 µm) into zeolite beads or hollow spheres of the same size constituted of an agglomeration of particles (0.5–3 μm) composed of pure silica MFI-type nanosheets, by using di-quaternary ammonium-type surfactant. This strategy seems transposable to several other pure silica zeolite structures. It provides a simple and efficient new synthesis way for obtaining zeolitic beads and hollow spheres of controlled size. The crystallization seems to begin at the outer surface of the beads allowing at higher treatment temperature the acceleration of the dissolution kinetic of the inner amorphous part of the beads to form hollow spheres. Both plain beads and hollow spheres of silicalite-1 showed to be promising for molecular decontamination in continuous flow by adsorbing n-hexane in high amount.

This strategy of pseudomorphic transformation of silica amorphous beads into zeolite beads or hollow spheres seems transposable to several other zeolite structures. It provides a simple and efficient one shot synthesis way for obtaining zeolitic beads or hollow spheres of controlled size without the use of binders for industrial applications.

## Figures and Tables

**Table 1:** Diameter of the particles and thickness of the nanosheets building the 20 µm silicalite-1 beads obtained by pseudomorphic transformation. Silicalite-1 beads are presented as a function of their temperature and duration of synthesis (B-T(°C)-t(days)).

	Particle Diameter^a^ (µm)	Nanosheet Thickness (nm)
**B110-2**	0.2	2
**B110-3**	0.3	2
**B110-5**	0.5	2
**B130-2**	0.6	2
**B130-3**	1.2	2
**B150-1**	0.8	4
**B150-2**	1.4	5
**B150-3**	1.8	7
**B150-4**	2.0	7
**B150-5**	2.8	10

^a^ Particles obtained by nanosheets assembly/agglomeration.

**Table 2:** Textural properties of the calcined materials synthetized by pseudomorphic transformation and of the parent amorphous silica spheres. Silicalite-1 beads are presented as a function of their temperature and duration synthesis (B-T(°C)-t(days).

	S_BET_^a^ (m^2^/g)	S_mic_^b^ (m^2^/g)	S_mes_^b^ (m^2^/g)	S_ext_ (m^2^/g)	V_tot_^c^ (cm^3^/g)	V_micro_^b^ (cm^3^/g)	V_meso_^b^ (cm^3^/g)	Mesopore Diameter ^d^ (nm)	Crystallization Degree (%)^e^
**Parent silica sphere**	545				1.04			3.4–6.4–10.7	0
**B110-2**	282	191	0	91	0.329	0.106	0	3.3–6.6–9.1	58
**B110-3**	354	246	0	108	0.357	0.137	0	2.3–3.3–8.6	76
**B110-5**	595	398	45	152	0.552	0.189	0.184	4.3	100
**B130-2**	458	327	31	100	0.377	0.155	0.135	4.7	86
**B130-3**	374	246	0	128	0.335	0.134	0.105	5	74
**B150-1**	352	251	0	101	0.302	0.138	0.087	3.4–5.4	76
**B150-2**	381	296	0	85	0.330	0.162	0.014	3.2–6.4	90
**B150-3**	395	284	0	111	0.326	0.157	0.078	3.4–5.5	87
**B150-4**	370	283	0	87	0.321	0.155	0.080	3.4–5.5	86
**B150-5**	390	296	0	94	0.305	0.162	0.063	3.4–5.5	90

^a^ Specific surface area determined by using BET (Brunauer–Emmet–Teller) method. ^b^ Determined from t-plots with corrections if needed according to Galarneau et al., Langmuir, 2014, 2018. ^c^ Total pore volume determined at the relative pressures p/p° = 0.99. ^d^ Determined from the pore size distribution obtained by DFT method applied on the adsorption branch of isotherm. ^e^ Crystallization degree= V_micro zeolitic_/V_micro zeolitic of reference MFI nanosheets_; V_micro zeolitic of reference silicalite-1_ = 0.18 cm^3^/g.

## Supplementary Materials

The following are available online at https://www.mdpi.com/1420-3049/25/11/2563/s1, Figure S1: t-plot for N_2_ adsorbed at −196 °C in B110-2, B110-3, B110-5, B130-2, B130-3, B150-1, B150-2, B150-3, B150-4, and B150-5.
