# Supplementary material for: Synthesis of Hierarchical Zeolites with Morphology Control: Plain and Hollow Spherical Beads of Silicalite-1 Nanosheets
Source: Molecules. 2020 May 31;25(11):2563. doi: 10.3390/molecules25112563 (PMC7321357; doi:10.3390/molecules25112563)
Supplement: Supplementary file 1 [file molecules-25-02563-s001.pdf]

molecules

MDPI

Supplementary Materials

Synthesis of Hierarchical Zeolites with Morphology Control: Plain and Hollow Spherical Beads of Silicalite-1 Nanosheets

Kassem Moukahhal [1,2,3], Bénédicte Lebeau [1,2], Ludovic Josien [1,2], Anne Galarneau [4], Joumana Toufaily [3], Tayssir Hamieh [3], and T. Jean Daou [1,2,*]

[1] University of Haute Alsace (UHA), CNRS, Axe Matériaux à Porosité Contrôlée (MPC), Institut de Science des Matériaux de Mulhouse (IS2M), UMR 7361, F-68093 Mulhouse, France; kassem.moukahhal@uha.fr (K.M.); benedicte.lebeau@uha.fr (B.L.); ludovic.josien@uha.fr (L.L.)

[2] University of Strasbourg (UniStra), F-67000 Strasbourg, France

[3] Laboratory of Materials, Catalysis, Environment and Analytical Methods Faculty of Sciences, Section I, Lebanese University Campus Rafic Hariri, Hadath, Lebanon; joumana.toufaily@ul.edu.lb (J.T.); tayssir.hamieh@ul.edu.lb (T.H.)

[4] ICGM, University of Montpellier, CNRS, ENSCM, Montpellier, France; anne.galarneau@enscm.fr

* Correspondence: jean.daou@uha.fr; Tel.: +00-(33)-389336739

To evaluate the micro- and mesopore volumes and surface areas of the different 20 µm silicalite-1 beads (plain or hollow spheres), the nitrogen adsorption isotherms of the samples were analyzed by the t-plot method (Figure S1) following the recommendations of A. Galarneau [1,2].

For the transformation of p/p_0 pressure into nitrogen layer thickness t, the following equations were used with t (Å):

$$0.009 < p/p° < 0.12 \quad (1)$$

$$t1 = 1.62973 + 76.4748\ (p/p°) - 2171.7914\ (p/p°)^2 + 41734.77357\ (p/p°)^3 - 465290.41181\ (p/p°)^4 + 2.72432\ 10^6\ (p/p°)^5 - 6.43708\ 10^6\ (p/p°)^6 \quad (2)$$

$$0.13 < p/p° < 0.60 \quad (3)$$

$$t2 = 3.07721 + 5.64019\ (p/p°) \quad (4)$$

$$0.60 < p/p° < 0.75 \quad (5)$$

$$t3 = 4592.05803 - 38117.31548\ (p/p°) + 131602.19741\ (p/p°)^2 - 241680.40239\ (p/p°)^3 + 249079.8569\ (p/p°)^4 - 136632.44762\ (p/p°)^5 + 31182.4149\ (p/p°)^6 \quad (6)$$

$$0.75 < p/p° < 0.90 \quad (7)$$

$$t4 = 2098.4 - 10711\ (p/p°) + 18954\ (p/p°)^2 - 9197.5\ (p/p°)^3 - 10624\ (p/p°)^4 + 14046\ (p/p°)^5 - 4553\ (p/p°)^6 \quad (8)$$

The corrections for the overestimation of the microporous volume [1] and the underestimation of the mesoporous + external surface areas [2] demonstrated for mechanical mixtures of zeolite (FAU-Y) and mesoporous material (MCM-41) were applied for the family of hierarchical silicalite-1, with following the formula:

(a) for micropore volume corrections:

$$\mathbf{(V_{mic}/V_{tot})_{tpt} < 12\%} \quad (9)$$

$$V_{mic\text{-}calc}/V_{mic\text{-}tpt} = 1 \quad (10)$$

$$\mathbf{12\% < (V_{mic}/V_{tot})_{tpt} < 46\%} \quad (11)$$

$$V_{mic\text{-}calc}/V_{mic\text{-}tpt} = 0.52947\ (V_{mic}/V_{tot})_{tpt}^{0.25334} \quad (12)$$

$$(V_{mic}/V_{tot})_{tpt} > 46\% \quad (13)$$

$$V_{mic\text{-}calc}/V_{mic\text{-}tpt} = 1.40 \quad (14)$$

(b) for mesopore + external surface areas corrections:

$$(V_{mic}/V_{tot})_{tpt} < 30\% \quad (15)$$

$$(S_{mes+ext})_{calc}/(S_{mes+ext})_{tpt} = 1 \quad (16)$$

$$30 < (V_{mic}/V_{tot})_{tpt} < 55\% \quad (17)$$

$$(S_{mes+ext})_{calc}/(S_{mes+ext})_{tpt} = 1.6 - 0.02138\ (V_{mic}/V_{tot})_{tpt} \quad (18)$$

$$(V_{mic}/V_{tot})_{tpt} > 55\% \quad (19)$$

$$(S_{mes+ext})_{calc}/(S_{mes+ext})_{tpt} = 0.38 \quad (20)$$

with $(V_{mic}/V_{tot})_{tpt}$ determined by t-plot analysis expressed in %.

V_{mic} was taken as the intercept with the y-axis of the low pressure fit of the t-plot and V_{tot} was taken as micropore + mesopore volume ($V_{mic+mes\text{-}tpt}$) as presented in Figure S1.

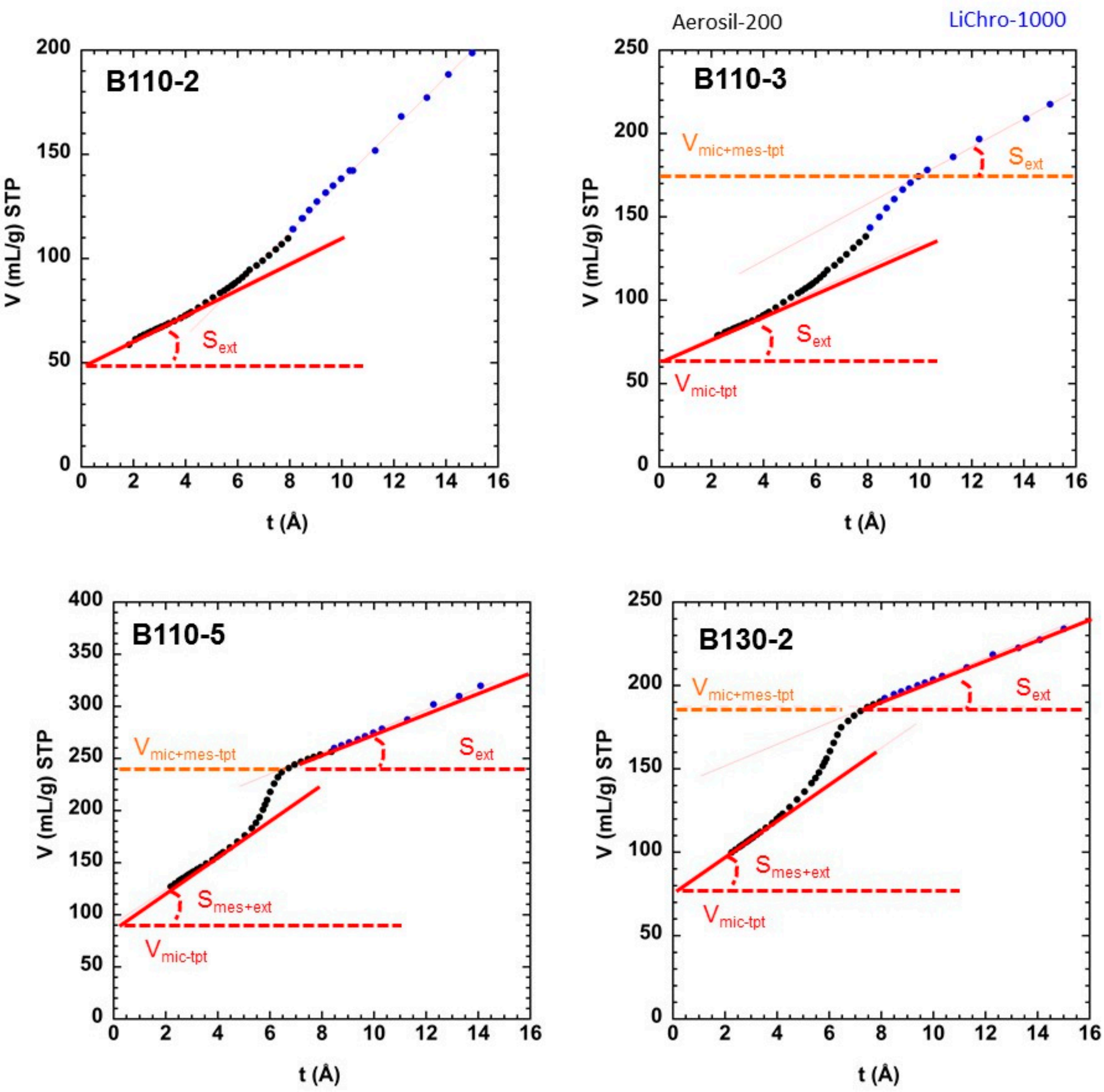

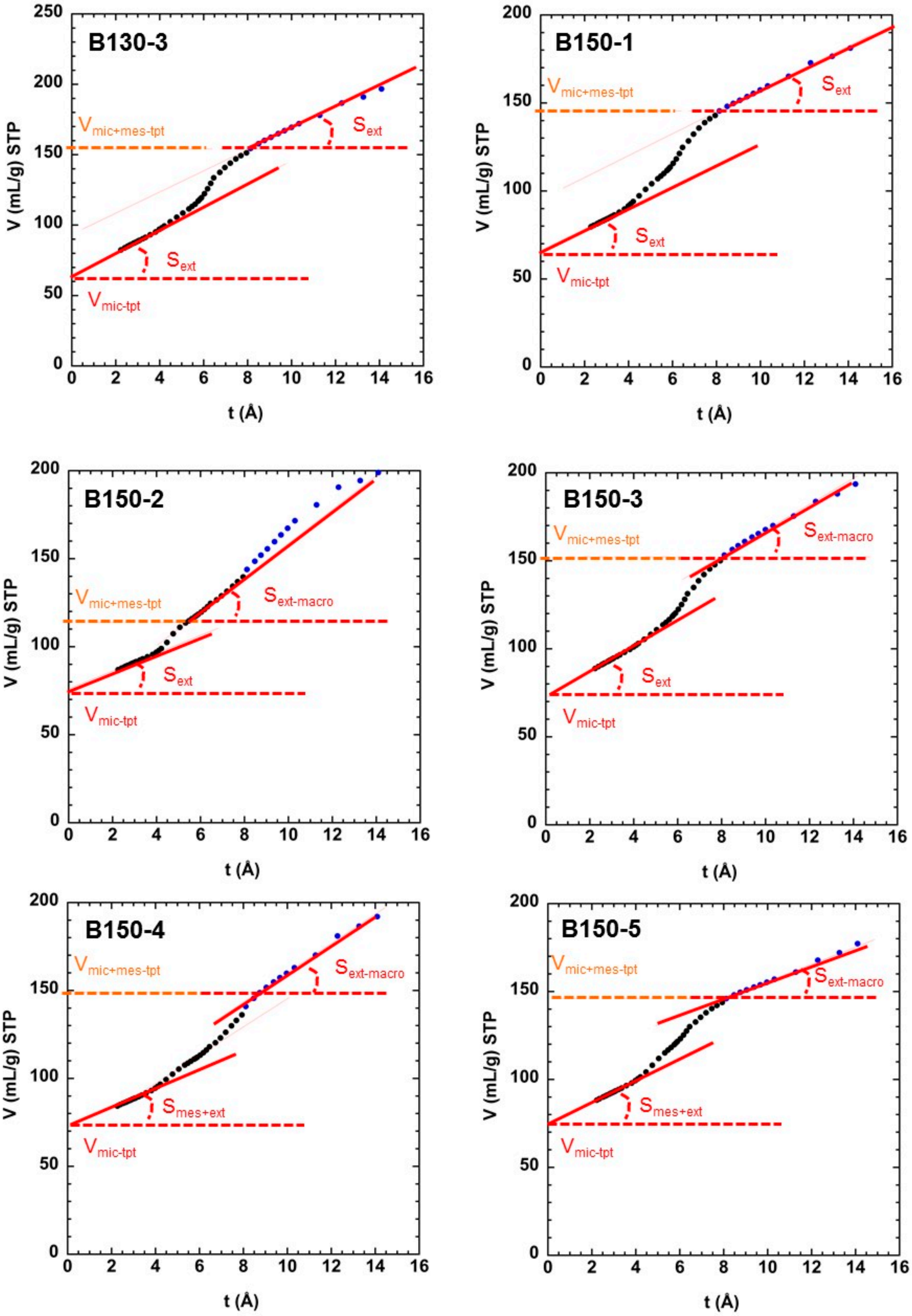

Figure S1. t-plot for N_2 adsorbed at 77 K in B110-2, B110-3, B110-5, B130-2, B130-3, B150-1, B150-2, B150-3, B150-4 and B150-5.

1 Galarneau, A.; Villemot, F.; Rodriguez, J.; Fajula, F.; Coasne, B. Validity of the *t-plot* Method to Assess Microporosity in Hierarchical Micro/Mesoporous Materials. *Langmuir* 2014, *30*, 13266-13274, doi:10.1021/la5026679.

2 Galarneau, A.; Mehlhorn, D.; Guenneau, F.; Coasne, B.; Villemot, F.; Minoux, D.; Aquino, C.; Dath, J.P. Specific Surface Area Determination for Microporous/Mesoporous Materials: The Case of Mesoporous FAU-Y Zeolites. *Langmui*r **2018**, *34*, 14134-14142, doi:10.1021/acs.langmuir.8b02144.
